# Retrieval and Monitoring Processes during Visual Working Memory: An ERP Study of the Benefit of Visual Semantics

**DOI:** 10.3389/fpsyg.2017.01080

**Published:** 2017-07-05

**Authors:** Elizabeth Orme, Louise A. Brown, Leigh M. Riby

**Affiliations:** ^1^Department of Psychology, Northumbria UniversityNewcastle upon Tyne, United Kingdom; ^2^School of Psychological Sciences & Health, University of StrathclydeGlasgow, United Kingdom

**Keywords:** event-related potentials, ERP, EEG, visuo-spatial working memory, visual short-term memory, memory retrieval, semantic memory, unitization

## Abstract

In this study, we examined electrophysiological indices of episodic remembering whilst participants recalled novel shapes, with and without semantic content, within a visual working memory paradigm. The components of interest were the parietal episodic (PE; 400–800 ms) and late posterior negativity (LPN; 500–900 ms), as these have previously been identified as reliable markers of recollection and post-retrieval monitoring, respectively. Fifteen young adults completed a visual matrix patterns task, assessing memory for low and high semantic visual representations. Matrices with either low semantic or high semantic content (containing familiar visual forms) were briefly presented to participants for study (1500 ms), followed by a retention interval (6000 ms) and finally a same/different recognition phase. The event-related potentials of interest were tracked from the onset of the recognition test stimuli. Analyses revealed equivalent amplitude for the earlier PE effect for the processing of both low and high semantic stimulus types. However, the LPN was more negative-going for the processing of the low semantic stimuli. These data are discussed in terms of relatively ‘pure’ and complete retrieval of high semantic items, where support can readily be recruited from semantic memory. However, for the low semantic items additional executive resources, as indexed by the LPN, are recruited when memory monitoring and uncertainty exist in order to recall previously studied items more effectively.

## Introduction

In the episodic memory domain, there is strong evidence for a range of ERP components related to familiarity (Curran, 2000; Smith et al., 2009), recollection (Wilding, 2000; Smith et al., 2009; Brown and Riby, 2013) and post-retrieval monitoring (Johansson and Mecklinger, 2003; Riby et al., 2008) during the retrieval of past events. There is increasing evidence suggesting some overlap in the network of processes involved during both long-term episodic and short-term (working) memory retrieval (e.g., Bundesen, 1990; Cabeza et al., 2002; Hellerstedt and Johansson, 2016). However, the interaction between these systems has largely been neglected in the working memory domain, with research tending to focus on processes within the working memory system. With more recent working memory models explicitly taking account of information flow to and from long-term memory (e.g., Baddeley, 2000, 2012; Logie, 2011), researchers are now increasingly addressing the mechanisms by which long-term memory may support working memory. In the present study, we manipulated the semantic content of to-be-remembered abstract visual material, and directly investigated the engagement of episodic retrieval mechanisms during visual working memory recall. Where visual semantic information is more readily available within the stimuli, episodic retrieval (the conscious recollection of a previous specific events and contextual information; Rugg and Vilberg, 2013) may be more freely or automatically engaged.

### Visual Working Memory and Multimodal Coding

From a vision science perspective, research has focused quite specifically on functioning within visual working memory in order to understand the processes that underlie the capacity to retain visual material over the short term (e.g., Luck and Vogel, 1997; Bays and Husain, 2008; Zhang and Luck, 2008; for review, see Luck and Vogel, 2013). This research typically uses abstract, simple feature and object stimuli (e.g., basic colors, shapes, orientations, etc.) in order to emphasize reliance upon temporary visual storage, and to limit input from other cognitive resources such as verbalization (Luck and Vogel, 2013; Ma et al., 2014). However, real-world context and ‘visual long-term memory’ are increasingly being explored in this literature (Brady et al., 2013; Ma et al., 2014). From a cognitive psychology perspective, working memory models have, for some time, explicitly identified a relationship between long- and short-term memory resources (e.g., Logie, 1995, 2011; Baddeley, 2000, 2012; Cowan, 2001, 2005), in addition to the opportunity for cross-domain processing within working memory (e.g., Logie, 2011; Baddeley, 2012). Yet, the processes involved in these relationships are not yet well understood, especially when considering long-term memory support of visual working memory, which can be considered a form of multimodal coding.

Memory is more successful when the stimulus is amenable to being encoded across different modalities. Within long-term memory, storage of both verbal and visual material benefits from the availability of both visual and verbal codes. For example, Paivio’s (1971, 1991) dual coding theory accounts for the finding that concrete words (e.g., ‘jacket’) are better recalled than abstract words (e.g., ‘jealous’), due to the imagery that is associated with concreteness. Memory for abstract visual stimuli is also superior when semantic, meaningful context is provided via additional verbal information (Bower et al., 1975; Santa, 1975; Verhaeghen et al., 2006). Thus, long-term memory performance varies according to the abstractness of the stimuli, and the information available at encoding. Regarding specifically visual working memory, Brown et al. (2006) showed that abstract visual stimuli, in the form of black and white matrix patterns (Phillips and Christie, 1977; Della Sala et al., 1999) are better recalled when the stimuli are more easily verbally recoded (see also Postle and Hamidi, 2007; Orme, 2009; Mammarella et al., 2014, for a matrix recognition task). Although the stimuli were simple matrix patterns, the participants’ verbal labels corresponded not only to relatively basic shapes or symbols (e.g., ‘the letter L,’ ‘a rectangle,’ and ‘a diamond’) but also to more elaborate, complex configurations (e.g., ‘a face,’ ‘steps,’ and animals), suggesting that the stimuli were being semantically elaborated. Brown and Wesley (2013) investigated the working memory components that underlie the effect, and found that the benefit associated with increased verbalization was not removed by articulatory suppression. They argued that, rather than explicit verbal recoding and rehearsal taking place within working memory, it is more likely that the source of the enhancement is the activation of semantic concepts (i.e., meaning; Postle et al., 2005; Orme, 2009; Darling et al., 2012).

Indeed, theoretically, it is argued that stored knowledge may be temporarily activated or explicitly drawn upon in order to support working memory capacity. For example, Logie’s (2011) workspace model states that information enters the working memory system via long-term stores, which can automatically activate relevant semantic knowledge. The novel and activated material is then stored in specialized temporary components and can be actively refreshed or manipulated, using executive processing resources. Executive resources could also be used to draw upon long-term knowledge more actively/strategically. Therefore, semantic activation may occur automatically at encoding, or strategically using executive resources (Logie, 2011). By considering participants’ reported strategy use after task completion, Brown and Wesley (2013) suggested that automatic semantic activation may indeed occur. Those participants who did not report actively using mixed (visual and verbal-related) strategies exhibited smaller capacity than those who did report using a mixed strategy, specifically in the low semantic task. Thus, the non-strategic group showed a disproportionately larger benefit of the high semantic stimuli, suggesting automatic activation of semantics (Riby and Orme, 2013). However, suppression of executive resources (using random spatial tapping) was also found to remove the semantic benefit. Therefore, it was additionally argued that even when semantics are automatically activated, executive processes can be used by actively encoding the semantics with the novel pattern configurations, actively combining semantic and novel material, and/or actively drawing upon the semantic context at recall (Mammarella et al., 2014). Furthermore, because the strategic group of participants was able to perform well in the low semantic task, it seems that there is a role for executive resources being used actively to seek out meaning (i.e., to encode the information more actively/strategically; see also Riby and Orme, 2013). Thus, overall, there appear to be at least two mechanisms by which high meaningfulness can enhance temporary memory for visual stimuli – by automatic activation of semantics, and by active strategy use.

In a similar context, Allen and colleagues investigated the beneficial effect of meaningful spatial layouts (a well-known keypad) for verbal working memory performance (Allen et al., 2015; see also, Darling and Havelka, 2010; Darling et al., 2012). Allen et al. showed that spatial interference during the encoding phase, but not the recall phase, removed the positive effect of spatial semantics. They argued that the critical phase for multimodal processing, then, is encoding. This leaves open the possibility that meaningfully encoded information is more readily accessible at recall, given that any required multimodal processing will, typically, already have taken place. Indeed, multiple component models of working memory suggest that conscious access to multimodal material stored in working memory may be achieved via an episodic buffer component (Baddeley, 2000, 2012; Baddeley et al., 2011; Logie, 2011). This buffer brings together material across working memory components, and between working memory and long-term memory. In the context of visual working memory performance, Hu et al. (2016) have recently described this buffer as resulting in a *privileged state* in working memory. It allows direct conscious access to stored information, but is also intimately related to both top-down (e.g., goal-directed prioritization) and bottom-up attention processes. Thus, emerging evidence suggests a key role for activated semantics to boost visual working memory capacity. Executive resources also seem to be intimately involved in visual working memory performance and, while executive input may be particularly important at encoding, less is known about the retrieval processes that could contribute to visual working memory recall when semantic context is manipulated.

### Event-Related Potential Correlates of Recollection and Retrieval Monitoring

The present study takes advantage of an event-related potential paradigm to investigate the potential involvement of episodic memory processes at the retrieval stage of a visual working memory task involving low and high semantic content. When studying retrieval of words in a typical old/new episodic memory paradigm, it has been proposed that processing new words primarily activates semantic memory, whereas successful retrieval of old words requires episodic memory. The latter of these results in an enhanced PE component between 400 and 800 ms (Friedman and Johnson, 2000). This component has been found to be enhanced when items are being consciously remembered (Smith, 1993), and when the encoding context is retrieved (e.g., Wilding and Rugg, 1996; Wilding, 2000), supporting the view that the PE component is an index of successful recollection.

In addition to successful recollection, two further components have been identified which index post-retrieval processes. The first of these is the late right-frontal effect which has been reported to reflect confidence in source judgements in old/new paradigms (Cruse and Wilding, 2009). However, of particular relevance to the current study, the aforementioned PE component is often observed in conjunction with a late posterior negativity (LPN), which is specifically enhanced following successful recognition of old items. The onset of this negative component immediately follows the participant’s response, and is sustained for some time. Johansson and Mecklinger (2003) reviewed studies in which this component is observed and concluded that the LPN is associated with two potential retrieval processes. The first of these is action monitoring, due to conflict during the recognition judgement. Secondly, at retrieval, the LPN may be generated as a result of the binding of sensory information and the use of imagery. More specifically, the amplitude of the component may be determined by how readily this representation is generated, and the individuals’ confidence in the memory decision. This suggests that the LPN may facilitate further examination of the memory representation, and validation of the response where uncertainty exists. In a more recent review, Mecklinger et al. (2016) discussed the localisation of the LPN, concluding that any topographical differences observed within and between studies is unlikely to be attributed to task modality, but rather the need for higher cognitive control processes in some paradigms. In such tasks, the distribution of the LPN may be more anterior. This highlights the importance of investigating the extent to which these processes may also be involved in working memory retrieval.

### Commonalities between Episodic Memory and Working Memory Retrieval

There has been some consideration of a potential overlap between episodic memory recollection and working memory processes. Cabeza et al. (2002) argued that there is clearly some differential fMRI activation observed within a fronto-parieto-cerebellar neural network. Nevertheless, episodic memory retrieval and working memory performance do elicit activation in some common brain regions, including bilateral superior parietal cortex, which they suggested is attention-related. Furthermore, Cabeza et al. (2002) showed that inferior parietal cortex was more greatly activated by working memory than episodic retrieval. Interestingly, however, Vilberg and Rugg (2008) concluded that inferior parietal cortex fMRI activity likely reflects the functioning captured by the ERP PE effect described above, and that both of these neural correlates are likely directly related to successful recollection in memory. Furthermore, they proposed that inferior parietal cortex activity may support the operation of the episodic buffer component discussed earlier (Baddeley, 2000, 2012; Logie, 2011) or that, at least, the region forms part of a network which produces the buffer’s functions. These conclusions strongly suggest a need for further research investigating the PE ERP component in the context of working memory performance.

Recently, Elward and Wilding (2010) showed a positive relationship between working memory capacity and the magnitude of the neural correlate of episodic retrieval (PE component) for targets, but not non-targets. Additionally, working memory capacity predicted the magnitude of the difference between the PE component for targets and non-targets. The authors concluded that this ERP effect may reflect online maintenance of information, and possibly cognitive control over prioritization of information in memory. Thus, episodic retrieval and working memory performance may both rely upon this process and, given the arguments discussed above, it is also possible to predict modulation of the parietal episodic ERP component by the extent of multimodal coding.

In a recent study by Hellerstedt and Johansson (2016), it was observed that when participants were presented with a category label and asked to generate a category member in a word-stem completion paradigm, the LPN component was present. Interestingly, the component was attenuated when successful retrieval occurred, and enhanced when participants were presented with impossible word-stems. This suggests that retrieval of semantic information may give rise to the LPN observed in conjunction with old/new episodic memory effects. In this specific example, Hellerstedt and Johansson proposed that the LPN may represent participants’ continued semantic retrieval attempts when successful retrieval is not immediately achieved. Mecklinger et al. (2016) concluded that this supports the view that the LPN reflects domain-general processes, which are present in both episodic and semantic memory tasks.

### Aims

The present study examines ERP components typically associated with episodic old/new retrieval effects, using a novel visual working memory paradigm. Employing a modified version of the visual matrix task (Orme, 2009; Riby and Orme, 2013), we manipulated the semantic content of to-be-remembered stimuli. Riby and Orme (2013) reported that high semantic visual matrix patterns give rise to ERP components associated with semantic processing at encoding, and in turn this scaffolding by semantic memory results in a reduction in visual information processing and subsequent memory load. As discussed above, Elward and Wilding (2010) proposed that the amplitude of the PE component may be an index of online maintenance of information and working memory capacity. If this is the case, the increase in visual information load observed for the low semantic patterns may result in an increase of the PE ERP component. Alternatively, if the magnitude of the PE effect is related to the quality of the memory trace, due to semantic elaboration, we would expect a higher amplitude for the high semantic patterns.

In addition, we anticipated that the low semantic matrix patterns will result in an enhancement of the LPN effect, due to an increased reliance on image reconstruction and uncertainty in the response, following the findings of Hellerstedt and Johansson (2016). Finally, it is proposed that an increased reliance on higher level control processes, necessitated by multimodal coding and strategy use in this complex working memory task (Brown and Wesley, 2013), may result in a more anterior presentation of this later component.

## Materials and Methods

### Participants

Fifteen right handed, native English speaking, University of Northumbria undergraduate students (seven females; mean age = 23.5 years) were recruited via a poster advertisement campaign at the Psychology Department. All participants had normal or corrected-to-normal vision and participated on a voluntary basis. Participants did not receive payment but were awarded course credit where appropriate. This study was carried out in accordance with the recommendations of University of Northumbria, Health and Life Sciences Ethics Committee with written informed consent from all subjects. All subjects gave written informed consent in accordance with the Declaration of Helsinki. The participant characteristics and methodology below have been previously described by Riby and Orme (2013) in their analysis of the memory encoding aspects of the dataset which make up a larger program of work.

### Stimuli

**FIGURE 1:**
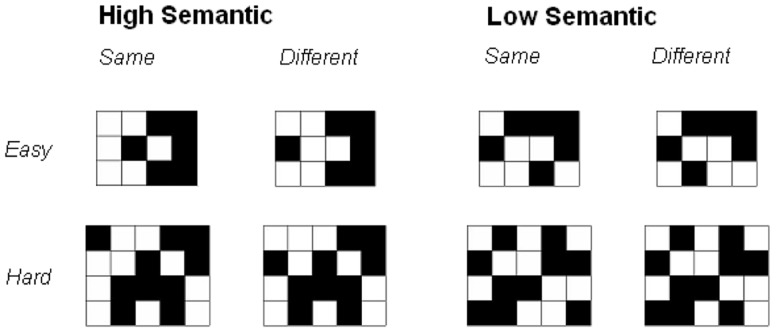
Examples of High and Low Semantic Stimuli across same/different and easy/hard trials.

The task utilized two sets of visual matrix patterns (Riby and Orme, 2013; **Figure 1**). The High Semantic set comprised patterns which are more easily represented semantically and are likely to readily elicit familiar visual forms. The High Semantic set were constructed with the aim of encouraging the processing of ‘pure’ visual representations (complete and coherent representation). Each pattern set consisted of twenty black and white grids for each level of complexity (defined as the number of cells in the pattern; ranging from 10 cells with 5 filled in black, to 26 cells with 13 filled). A set of distracter patterns was employed for the recognition test, providing a ‘different’ version of each matrix; this differed from the original by a single square being moved by one cell. It is important to note the low versus high semantic stimuli has previously been verified to differ in meaningfulness and semantic content but matched for structural complexity (Orme, 2009; also Riby and Orme, 2013 for comprehensive discussion of high versus low stimulus selection and matching procedures). The stimuli ranged from 3 to 5 cm in height. At a viewing distance of 55 cm, stimuli were presented with a visual angle of 3.12–5.25°.

### Procedure

**FIGURE 2:**
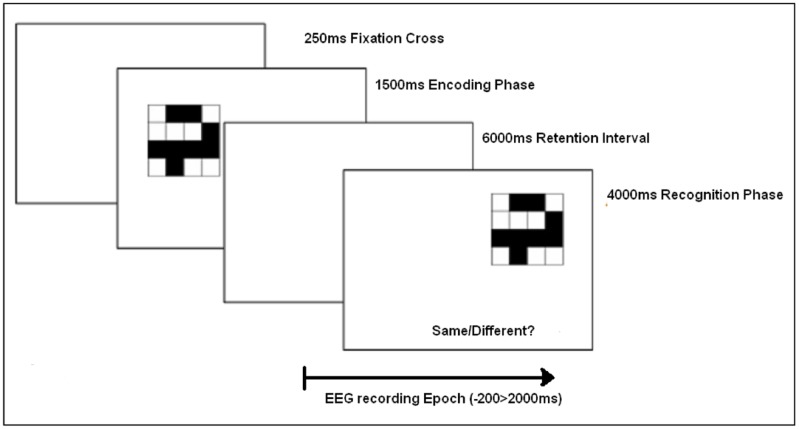
Task protocol for each encoding/recognition experimental trial.

Each trial consisted of a 250 ms fixation cross on the computer screen, followed by the memory encoding array of a single visual pattern, presented for 1500 ms. After a retention interval of 6000 ms, the recognition test array was presented. The recognition test pattern offset from the initial encoding stimulus to prevent retinotopic overlap. The offset was achieved by mapping four locations on the screen (1) upper left, (2) upper right, (3) lower left, and (4) lower right (spaced by approximately two stimulus lengths). The recognition test array was randomly relocated to one of these positions as illustrated in **Figure 2**. Participants were then asked to make a recognition judgment as to whether the test pattern was the same (‘old’) or different to the encoded stimuli by pressing the ‘Z’ or ‘M’ keys, respectively (counterbalanced across subjects). The next trial began 4000 ms after the onset of the test stimuli.

After verbal instructions, four practice trials were completed by the participants. In the experimental session, participants completed 360 randomized trials split into four blocks (90 trials per block). The 360 trials comprised 20 high semantic trials and 20 low semantic trials at all nine levels of complexity. In half of the trials a same (‘old’) response was required, while in the other half of the trials a different (‘new’) response was required. In ‘different’ trials the study and test patterns differed by one cell. The stimuli were presented within an array of 320 mm × 210 mm, and participants were seated to ensure a viewing distance of approximately 55 cm.

### EEG Acquisition

EEG was recorded from 32 channels using an electrode cap (Biosemi) based on the international 10–20 system (Jasper, 1958). All EEG recordings were referenced to linked mastoids. To assess eye blinks, electrodes were placed above and below the left eye to record the electrooculogram. All signals were digitized at a rate of 2048 per second with a recording epoch of -200 to 2000 ms. ERPs were time locked to the onset of the test stimulus during the retrieval phase of the task. Automatic eye-blink correction, artifact rejection (rejection criterion: -75 μV to +75 μV) and ERP averaging were conducted offline using NeuroScan Edit 4.3.

In order to be consistent with previous research on episodic memory retrieval, epochs containing correct same (‘old’) judgements were included for analysis. A minimum trial epoching approach was used with 16 trials set as the minimum for inclusion in the average of interest. There were 45 trials entered (range 16–75) and 39 (range 18–84) on average for the low and high semantic task, respectively. The measurement intervals were selected on the basis of visual inspection of the ERPs and time intervals reported elsewhere (e.g., Wilding, 2000; Riby et al., 2008). An estimate of the area under the curve (AUC) was calculated for each of the time windows described below (for discussion of the analytical strategy for ERP amplitude data, Luck, 2005).

## Results

### Analytical Strategy

**FIGURE 3:**
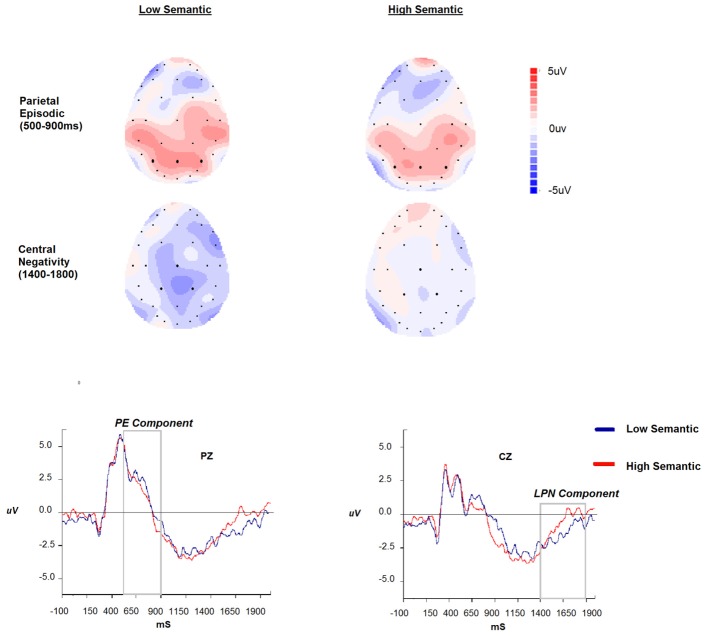
The parietal episodic and central negativity scalp maps across stimulus type (high vs. low semantic), ERPs and time windows (500–900 ms and 1400–1900 ms) at selected Pz and Cz sites.

The regions of interest were selected based on visual inspection and consultation of the literature. The PE memory effect (e.g., Friedman and Johnson, 2000, for review) is typically observed over parietal regions in the 400–800 ms time region and for verbal material left lateralised. Due to the nature of the stimuli (visual rather than verbal) and the paradigm (working rather than episodic memory), a 2 (Stimulus: Low versus High Semantic) × 3 (Region: left, central, right) ANOVA was conducted on AUC data for the early parietal positivity (P3, Pz, P4; 500–900 ms). The LPN was our second component of interest with the selection again based on visual inspection and the aforementioned work by Mecklinger et al. (2016). We anticipated the LPN to be elicited when there is uncertainty whilst recovering difficult to remember low semantic items, but due to the nature of the stimuli we anticipated that the component may be more anterior than typically observed. This is confirmed in **Figure 3** where the component is centered around central parietal electrodes. Therefore, a 2 (Stimulus: Low versus High Semantic) × 3 (Region: left, central, right) ANOVA was conducted on AUC data for the LPN (CP1, Cz, Cp2; 1400–1800 ms). Subsequent data analyses considered behavioral performance and how the magnitude of the components related to the response time and accuracy during the tasks.

The analysis of the PE component showed no significant effect of stimulus type or region (*p* > 0.05), and the interaction between stimulus type and region was not significant (*p* > 0.05). For the LPN component the main effect of stimulus type was significant {*F*(1,14) = 3.41, *p* = 0.08; $\eta_{p}^{2}$ = 0.19; with two extreme values [greater than three times the interquartile range] removed *p* = 0.006; $\eta_{p}^{2}$ = 0.47}. The main effect of region, and the interaction with stimulus type, were not significant (all *p* > 0.05). These data, illustrated in **Figure 3**, demonstrate the expected central negativity, with the magnitude being greater for the low semantic stimuli. For completeness, and to be consistent with previous research, we repeated the analysis on parietal electrodes where previous research has identified the effect. There was a main effect of region [*F*(2,28) = 4.2, *p* < 0.05; $\eta_{p}^{2}$ = 0.23] demonstrating greater negativity at the Pz location compared to P3 and P4. The effect of stimulus type and interaction with region was not significant *p* > 0.05.

The behavioral data described in detail elsewhere (Riby and Orme, 2013; **Table 1**) revealed more accurate (79.3% vs. 71.9%) and quicker responses (1443 ms vs. 1529 ms) for correct ‘old’ judgements in the high semantic stimulus condition relative to the low semantic condition, indicative of superior memory recall. To aid in the interpretation of the ERP data (particularly the LPN since the functional significance is unclear), correlation analyses were also carried out between these data (response time and accuracy) and the magnitude of the early PE and LPN ERP effects. No correlations were significant between response time or accuracy and the early PE memory component (*p* > 0.05). For the LPN, however, response times for correctly recognizing low and high sematic patterns were consistently negatively correlated with the associated low and high semantic ERPs – CP1, Cz and CP2 AUC (**Table 1**). These relationships are of particular interest as a slower response time in either condition, and an associated higher magnitude LPN, is suggestive of additional processing mechanisms being engaged during task completion.

**Table 1:** The relationships between task response times and magnitude of the LPN ERP component at selected sites across low and high semantic stimulus types.

	Low semantic ERPs						High semantic ERPs					
	CP1		CZ		CP2		CP1		CZ		CP2	
	*r*	Sig. *p*	*r*	Sig. *p*	*r*	Sig. *p*	*r*	Sig. *p*	*r*	Sig. *p*	*r*	Sig. *p*
Low semantic RT	-0.16	0.28	-0.37	0.08	-0.32	0.12	-0.23	0.21	-0.45	<0.05	-0.45	<0.05
High semantic RT	-0.30	0.14	-0.46	<0.05	-0.40	0.07	-0.30	0.14	-0.49	<0.05	-0.49	<0.05

## Discussion

The aims of the present study were to explore known ERP components related to successful retrieval of episodic memories within a visual working memory paradigm. The findings demonstrated the differential engagement of memory retrieval processes depending on the semantic content of the stimuli presented at encoding. We also investigated whether relatively ‘pure,’ or domain-specific visual short-term recall was achieved or whether additional mechanisms, such as attentional control or multimodal coding in the form of semantic memory input, were engaged to support successful remembering. For the more easily remembered high semantic patterns, retrieval of the items proceeded in a relatively automatic manner due to the richness, unitized or more complete nature of the memory representation formed at encoding. However, during retrieval of the low semantic patterns, uncertainty may have existed when making a response. Consequently, additional resources were recruited to aid recall. Overall, the data is in line with Riby and Orme’s (2013) suggestion that if visual information is pieced together and unitized (or chunked) into a coherent whole, subsequent recall proceeds in a relatively automatic manner.

Consider first the episodic memory component. Early work on the episodic memory ERP effects demonstrated larger amplitude ERPs elicited by the recognition of previously recognized items accompanied by ‘remember’ responses than for those receiving a ‘know’ response (e.g., Smith and Guster, 1993) and when memory for an event includes contextual or source information (e.g., Wilding and Rugg, 1996). Together these episodic memory studies suggest the richer the memory created, the larger the magnitude of the PE effect. Similarly, and mirroring the work presented here, in the verbal domain encouraging the use of semantic memory strategies at encoding by employing a levels of processing paradigm aids later recall and increases the engagement of the PE memory component (e.g., Paller and Kutas, 1992; Weyerts et al., 1997). We therefore predicted greater magnitude ERPs whilst recalling high semantic patterns. The location and amplitude of the ERP components were consistent with previous research using verbal stimuli (e.g., Brown and Riby, 2013). However, there is an important deviation from studies examining verbal episodic memory where there are ‘new’ items to consider in the retrieval phase rather than ‘similar’ items examined here. In those studies, a comparison across ‘old’ and ‘new’ items (or a subtraction is preformed), alongside the critical experimental condition, to more precisely isolate the memory processes engaged. As such, a note of caution is warranted in the identification of the observed ERP as the PE component. Regardless, although performance was enhanced when retrieving high semantic patterns, examination of the ‘raw’ amplitude of the ERP component revealed similarity for the two stimulus types. These finding do not seem compatible with our original predictions. Allen et al. (2015) provided evidence that meaningful multimodal coding may involve processes occurring at the encoding phase (see also Riby and Orme, 2013). This perhaps leaves the same processes to be employed in retrieval of the two stimulus sets and a richer memory representation being more readily available for the high semantic stimuli (indexed by behavioral performance). In the present data, it seems that both stimulus types equivalently trigger the observed ERP component. However, we can use the response times and accuracy as behavioral measures of the efficiency of memory recall. Faster responses and superior recall for the high semantic patterns is suggestive of a relatively automatic engagement of the underlying memory processes for the newly unitized stimuli. For the low semantic patterns, longer responses times suggest less efficient use of these processes, due to the fragmented nature of the stimuli and possibly due to the need to draw on resources associated with image reconstruction, and memory search in order to validate the response at retrieval, as indexed by the LPN observed in the 1400–1800 ms epoch. These findings are in line with ERP episodic memory studies where they have used response time and accuracy as measures of superior and more efficient recall. Indeed, Rhodes and Donaldson (2007) observed superior recall for more unitized representations, equivalence in the PE effect, mirroring work here, and differential engagement of retrieval mechanisms (PE vs. bi-lateral frontal effects in the examination of dual process accounts of memory) dependant on the unitized nature of the stimuli. These data should be treated with caution but demonstrate the worth of traditional behavioral measures of effort and efficiency (reaction times and accuracy) in the interpretation of ERP data and clarifying the functional significance of components. The findings here warrant further investigation of the observed ERP component to identify exactly which underlying memory processes are triggered at retrieval.

The observed LPN suggests differential engagement of later processing mechanisms supporting visual working memory. For the high semantic patterns, Riby and Orme (2013) presented evidence that the encoded memory representation is richer, as evidenced by enhanced P300 and N400 components. The authors interpret this as evidence of more efficient unitization (Rhodes and Donaldson, 2008), where pre-existing semantic knowledge can scaffold memory and create more complete representations. It is proposed that the availability of semantic support and the unitization of the memory representation, ‘pure’ recall (the engagement of controlled processes not required) was evident for the high semantic patterns. As discussed above, Johansson and Mecklinger (2003) report that the LPN is attenuated in cases where the memory representation is more readily generated and where there is high memory confidence. In addition, Hellerstedt and Johansson (2016) showed that successful semantic retrieval also results in a reduction to this component.

When considering the low semantic patterns, the opposite inference can be made. The enhanced LPN may highlight the engagement of differential retrieval and post-retrieval processes or attentional control mechanisms may be needed to guide successful remembering. Riby and Orme (2013) presented evidence that, at encoding, the low semantic patterns lead to greater information load, and less activation of semantic memory, suggesting less efficient unitization of the to-be-remembered stimulus. It therefore follows that, at retrieval, the retrieved memory representation is less ‘pure,’ incomplete and more complex in nature. As a result of this, the LPN may be enhanced due to the need for online reconstruction of the originally encoded stimulus, in order to examine and make a comparison with the test stimulus presented in the face of response uncertainty (Mecklinger et al., 2016), as well as an increased necessity for memory search processes to reference the image in semantic memory (Hellerstedt and Johansson, 2016). It is worthwhile noting the argument that, the LPN largely reflects post-retrieval processing mechanisms involving memory monitoring elicited by the uncertainty that continues after a response has been made, is the favored interpretation here given the overlap between response time and the activation of the LPN and our subsidiary analysis examining the relationship between the LPN and response time. Although further work considering the two accounts is warranted, if additional memory related and image reconstruction is necessary the onset of the LPN component would be expected before a response has been made in the retrieval phase.

In the present study, there are topographical differences in the presentation of the LPN, with a more anterior presentation. However, the nature of the tasks, and the timing of the negative component support the view that it is generated by the same underlying processes. Indeed, Brown and Wesley (2013) suggested that visual patterns task performance may involve more executive processing in cases where multimodal binding and a higher degree of strategy use is necessary. However, Brown and Wesley were unable to show at which stage in their task the executive demand became apparent, and the current data (as well as Riby and Orme, 2013) suggest that the encoding stage is likely to be most important, at least with this recognition task. Also, given the topographical differences between the more central later component observed here and the well-established LPN, we carried out a subsidiary analysis on RT and the magnitude of the LPN. We observed that for both stimulus types, greater amplitudes of the LPN were associated with slower response times. This directly implicates response uncertainty (regardless of whether recalling low or high semantic patterns) and increased decision time in the enhancement of the component. Therefore, it is proposed that the LPN observed here using a working memory paradigm is indeed reflective of post-retrieval processing mechanism previously associated with the LPN elsewhere (episodic memory; Mecklinger et al., 2016).

The LPN has been proposed to be associated with a range of possible post-retrieval processes. However, a further question that needs to be asked is precisely which processes are implicated here. Much of the previous research into the LPN focusses on episodic retrieval tasks, however, a small number of studies have also linked the component to semantic memory (e.g., Bai et al., 2015; Hellerstedt and Johansson, 2016). The novelty of the current project is that we extend this to working memory. Mecklinger et al. (2016) proposed that this potential link could reflect more general processes. These include the generation of memory representations and the integration of potentially linked items in semantic memory, and the comparison of such generated ‘matches’ with the retrieval stimulus. The LPN would then be attenuated in cases where a suitable match is found and retrieval is deemed successful. The task used here adds to the limited research into semantic retrieval, further supporting this argument. Indeed, the investigation has emphasized that further research is warranted and it is possible to use the groundwork provided here to investigate the processes overlapping between working memory and episodic memory.

Our previous work with this stimulus set has highlighted some potential limitations to the paradigm (Riby and Orme, 2013). Work by Orme (2009) demonstrated that the high and low semantic stimulus sets do not differ in terms of their physical complexity (such as the presence of symmetry, and the number of chunks of visual information). Furthermore, the fact that the observed performance advantage is not eliminated by verbal interference suggests that simple verbal labeling of the pattern elements cannot fully account for the effects observed (see also Brown and Wesley, 2013). Therefore, there is converging evidence that the high semantic pattern set is indeed supported by semantic memory. However, it is unclear if such representations are visual or verbal in nature, and the precise mechanisms by which semantics are integrated into working memory representations remain unclear. Brown and Wesley (2013) provided support for the role of executive resources in recall of visual matrix stimuli, but also demonstrated that to some extent semantic memory can be activated automatically. It is possible that this is achieved via the episodic buffer in working memory, which could allow direct conscious access to multimodal stimuli currently within the focus of attention, while also allowing for the deployment of controlled attentional resources, for example to draw upon strategic processes (Allen et al., 2014; Hu et al., 2016).

## Conclusion

Riby and Orme (2013) used the same task as was used presently to conclude that the enhanced memory performance observed for the high semantic patterns is achieved by allowing the effective unitization of short-term and long-term information resulting in a simplification of the memory representation and a reduction in overall memory load. The results of this study reinforce this view, by demonstrating that the low semantic patterns result in more complex and time consuming retrieval processes. This novel paradigm adds valuable insight into the nature of these post-retrieval processes in working memory tasks. Specifically, we add to the growing body of evidence that suggests the LPN has perhaps two roles when the stimuli are not readily retrieved. In this study when items to be recalled are not effectively ‘unitized’ or ‘chunked’ into a coherent whole after semantic support, resources are needed to aid in the binding and reconstruction of an item at retrieval. However, and due to the onset (overlapping response times) and prolonged nature of the LPN, these data largely reflect executive and monitoring processes that are engaged when uncertainty exists after the retrieval of ‘poorly’ encoded items into working memory.

## Author Contributions

LR and EO designed and were responsible for the day to day running of the project. LR, EO, and LB provided the intellectual input during the preparation of the manuscript. LR and EO were responsible for the data processing with the additional contribution of LB during analysis. All authors contributed significantly to the final version of the manuscript.

## Conflict of Interest Statement

The authors declare that the research was conducted in the absence of any commercial or financial relationships that could be construed as a potential conflict of interest.
